# Lack of Pannexin 1 Alters Synaptic GluN2 Subunit Composition and Spatial Reversal Learning in Mice

**DOI:** 10.3389/fnmol.2018.00114

**Published:** 2018-04-10

**Authors:** Ivana Gajardo, Claudia S. Salazar, Daniela Lopez-Espíndola, Carolina Estay, Carolina Flores-Muñoz, Claudio Elgueta, Arlek M. Gonzalez-Jamett, Agustín D. Martínez, Pablo Muñoz, Álvaro O. Ardiles

**Affiliations:** ^1^Departamento de Patología y Fisiología, Facultad de Medicina, Universidad de Valparaíso, Valparaíso, Chile; ^2^Centro Interdisciplinario de Neurociencia de Valparaíso, Facultad de Ciencias, Universidad de Valparaíso, Valparaíso, Chile; ^3^Escuela de Tecnología Médica, Facultad de Medicina, Universidad de Valparaíso, Valparaíso, Chile; ^4^Centro de Investigaciones Biomédicas, Escuela de Medicina, Universidad de Valparaíso, Valparaíso, Chile; ^5^Institute for Physiology I, University of Freiburg, Freiburg, Germany; ^6^Programa de Farmacología Molecular y Clínica, Instituto de Ciencias Biomédicas (ICBM), Facultad de Medicina, Universidad de Chile, Santiago, Chile; ^7^Center for Applied Neurological Sciences, Faculty of Medicine, Universidad de Valparaíso, Valparaíso, Chile; ^8^Centro Interdisciplinario de Estudios en Salud, Facultad de Medicina, Universidad de Valparaíso, Viña del Mar, Chile

**Keywords:** Pannexin 1, long-term depression, GluN2 subunits, behavioral flexibility, synaptic plasticity

## Abstract

Long-term potentiation (LTP) and long-term depression (LTD) are two forms of synaptic plasticity that have been considered as the cellular substrate of memory formation. Although LTP has received considerable more attention, recent evidences indicate that LTD plays also important roles in the acquisition and storage of novel information in the brain. Pannexin 1 (Panx1) is a membrane protein that forms non-selective channels which have been shown to modulate the induction of hippocampal synaptic plasticity. Animals lacking Panx1 or blockade of Pannexin 1 channels precludes the induction of LTD and facilitates LTP. To evaluate if the absence of Panx1 also affects the acquisition of rapidly changing information we trained Panx1 knockout (KO) mice and wild type (WT) littermates in a visual and hidden version of the Morris water maze (MWM). We found that KO mice find the hidden platform similarly although slightly quicker than WT animals, nonetheless, when the hidden platform was located in the opposite quadrant (OQ) to the previous learned location, KO mice spent significantly more time in the previous quadrant than in the new location indicating that the absence of Panx1 affects the reversion of a previously acquired spatial memory. Consistently, we observed changes in the content of synaptic proteins critical to LTD, such as GluN2 subunits of N-methyl-D-aspartate receptors (NMDARs), which changed their contribution to synaptic plasticity in conditions of Panx1 ablation. Our findings give further support to the role of Panx1 channels on the modulation of synaptic plasticity induction, learning and memory processes.

## Introduction

N-methyl-D-aspartate receptor (NMDAR)-dependent long-term potentiation (LTP) and long-term depression (LTD) are two opposing forms of activity-dependent synaptic plasticity which have emerged as putative cellular mechanisms underlying learning and memory in the central nervous system (CNS; Lynch, 2004; Collingridge et al., 2010). Whereas a plethora of studies support the role of LTP in memory related behaviors, data backing a significant participation of LTD in memory formation is currently sparse. Interestingly, growing evidence suggest that during complex memory related tasks requiring a high degree of behavioral flexibility information storage critically depends in LTD (Manahan-Vaughan and Braunewell, 1999; Zeng et al., 2001; Kemp and Manahan-Vaughan, 2004; Morice et al., 2007; Nicholls et al., 2008; Dong et al., 2013; Liu et al., 2014). In the CA1 area of the hippocampus, a region actively involved in the formation and retrieval of memories, two predominant forms of LTD can be found, NMDAR- and metabotropic glutamate receptor (mGluR)-dependent LTD (Malenka and Bear, 2004; Collingridge et al., 2010). Although these LTD types involve different induction and signal transduction cascades, basically both share a common expression mechanism, namely the removal of postsynaptic AMPA receptors due to modifications in membrane trafficking (Collingridge et al., 2010).

Pannexin 1 (Panx1) is a membrane protein that forms non-selective channels (Bao et al., 2004). The protein is expressed by different cell types of the CNS (Vogt et al., 2005; Huang et al., 2007) and interestingly is enriched in postsynaptic densities from hippocampal and cortical neurons (Zoidl et al., 2007). Recent studies have revealed a novel physiological role of Panx1 channels in modulating neuronal excitability and plasticity (Prochnow et al., 2012; Ardiles et al., 2014). Transgenic animals lacking the Panx1 gene display an enhanced hippocampal LTP accompanied by behavioral alterations including increased anxiety, impaired object recognition and spatial memory deficits (Prochnow et al., 2012). Similarly, we have reported that mice lacking Panx1 protein showed an increased NMDAR-dependent LTP at the Schaffer-collateral CA1 pyramidal cell synapse whereas NMDAR-dependent LTD was abolished specifically in adult mice (Ardiles et al., 2014). These results could be replicated by pharmacological block of Panx1, indicating that this protein might regulate the sliding threshold for excitatory synaptic plasticity (Ardiles et al., 2014).

In despite of the importance of LTD and LTP in memory formation, the mechanisms by which Panx1 regulates plasticity remain unclear. In the present study, we investigated the molecular substrates of Panx1 modulation of plasticity. We have found that the absence of Panx1 modifies the expression of NMDARs in a subtype-specific manner, and influences the contribution of these receptors in LTP and LTD. Moreover, we demonstrate that consistent with a deficit in LTD expression, Panx1 knockout (KO) animals have a deficit in spatial reversal learning, supporting novel functions of Panx1 channels in synaptic plasticity and memory flexibility.

## Materials and Methods

### Animals

All experiments were carried out in 6–8 months old C57BL/6 or Panx1-KO mice. The generation of KO mice has been described previously (Anselmi et al., 2008). Mice were housed at 22°C at constant humidity (55%), 12/12 h dark–light cycle, with a light phase from 08:00 AM to 08:00 PM. Food and water were provided *ad libitum*. The use and care of the animals were approved by the Ethics and Animal Care Committee of Universidad de Valparaíso (BEA064-2015).

## Experimental Design

Animals were submitted during 31 consecutive days to behavioral tests. At the end of this period, mice were sacrificed to obtain hippocampal slices to study synaptic transmission and plasticity. Finally, after completion of the electrophysiological experiments, hippocampal slices were immediately frozen for biochemistry or histological studies.

### Behavioral Tests

Spatial working memory was assessed in a T-maze using a Delayed non-match to place (DNMTP) paradigm during 14 days with four trials per day and 15 min of intertrial delay. The T-maze apparatus was made of black Plexiglas (two goal arms of 10 × 20 cm and one start arm of 10 × 30 cm with a central partition at the end separating the goal arms (Deacon and Rawlins, 2006). Mice were food deprived using a diet consisting of a 15% of reduction of the regular feeding volume. During training and testing trials mice were rewarded with food pellets (45 mg dustless precision pellets, Bio-Serv, Frenchtown, NJ, USA) mixed with condensed milk. On each trial, both goal arms were baited with reward. Trials (60 s each one) consisted in a forced run in which one arm of the maze was blocked, followed by a choice run in which both arms were open (Figure 5E). A correct choice was scored when the animal entered the previously blocked goal arm to find the remaining reward. Spatial learning flexibility was assessed in a modified Morris water maze (MWM) to evaluate the forward and reverse spatial learning (Nicholls et al., 2008). The MWM consisted in a circular pool of white Plexiglas (120 cm diameter and 60 cm high) surrounded by distal extra-maze visual cues and a circular platform (10 cm diameter) used as the goal platform. Room and water temperature were held at 22–23°C. For first 2 days (days 1–2), the animals learned to swim to the visible platform which was randomly located in a different quadrant for each trial. The next 10 days (days 3–12), the animals learned to find a hidden platform localized at a fixed quadrant (acquisition phase). Finally, during the last 5 days (days 13–17), the animals needed to learn the position of a hidden platform located at the opposite quadrant (OQ) than the one used during the acquisition phase (reversal learning phase; Figure 5A). For all phases, four trials of 60 s were given each day at 15-min inter-trial intervals. During the last day of acquisition and reversal phases, the platform was removed from the pool to assess retention of the previously acquired information. Path, latency to find the platform and time spent in the quadrants was determined. All behavioral experiments were performed and analyzed blind to the genotype of each mouse.

**Figure 1:**
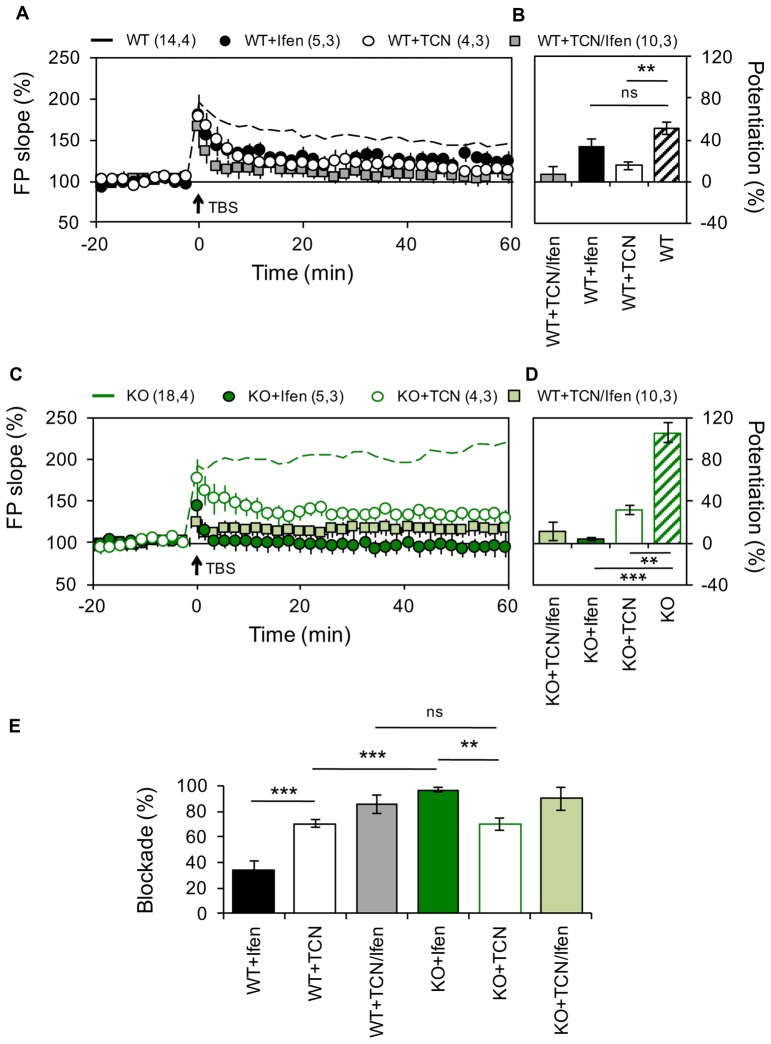
Altered contribution of GluN2 subunits to N-methyl-D-aspartate receptor (NMDAR)-dependent long-term potentiation (LTP) in the Schaffer Collateral–CA1 pathway from Pannexin 1 (Panx1)-knockout (KO) mice. **(A)** LTP obtained in slices from wild type (WT) in the absence (WT, dashed black line) or the presence of Ifenprodil (WT+Ifen, black circle), TCN-201 (WT+TCN, open circle) or TCN-201 plus ifenprodil (WT+TCN/Ifen, gray square). TBS protocol was delivered at the time indicated by the arrow. **(B)** Averaged LTP magnitude during the last 10 min of recording for WT (patterned bar), WT+Ifen (black bar), WT+TCN (open bar) and WT+TCN/Ifen (gray bar). One-way ANOVA (*F*_(3,24)_ = 17.35, *p* < 0.0001) followed by Dunnett’s *post hoc* test vs. WT. **(C)** LTP obtained in slices from Panx1-KO in the absence (KO, dashed green line) or the presence of Ifenprodil (KO+Ifen, green circle), TCN-201 (KO+TCN, open circle) or TCN-201 plus ifenprodil (KO+TCN/Ifen, light green square). **(D)** Averaged LTP magnitude during the last 10 min of recording for KO (patterned bar), KO+Ifen (green bar), KO+TCN (open bar) and KO+TCN/Ifen (light green bar). One-way ANOVA (*F*_(2,24)_ = 20.21, *p* < 0.0001) followed by Dunnett’s *post hoc* test vs. KO. **(E)** Percentage of LTP blockade for WT and KO slices in the presence of GluN2 antagonists. One-way ANOVA (*F*_(3,15)_ = 34.99, *p* < 0.0001) followed by Tukey’s *post hoc* test. The values in parentheses indicate the number of hippocampal slices (left) and the number of animals (right) used. **p < 0.01; ***p < 0.001; ns, non-significant.

**Figure 2:**
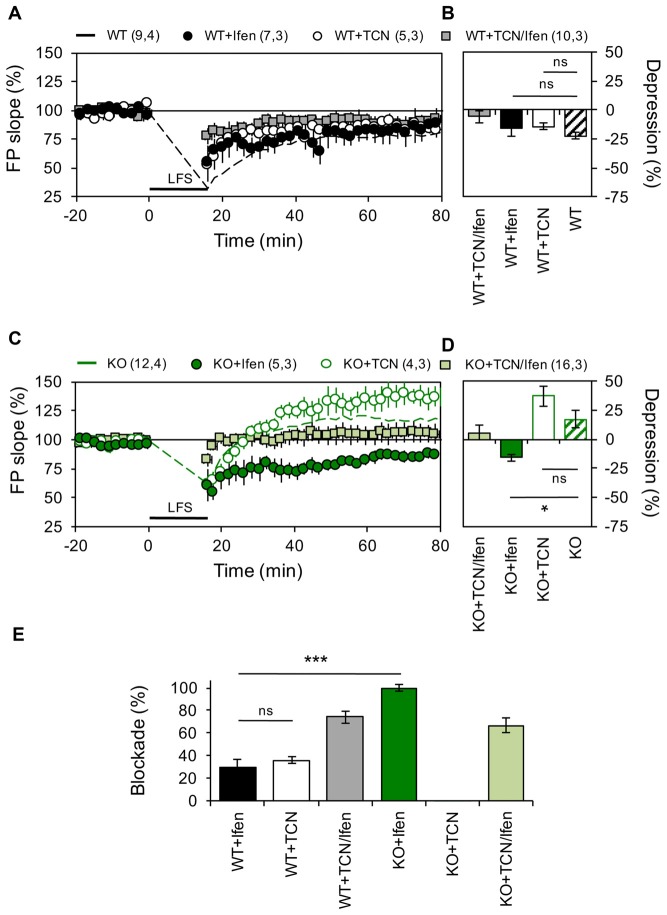
Panx1 deficiency modifies the contribution of GluN2 subunits to NMDAR-dependent long-term depression (LTD). **(A)** LTD obtained in slices from WT in the absence (dashed line) or the presence of Ifenprodil (WT+Ifen, black circle), TCN-201 (WT+TCN, open circle) or TCN-201 plus ifenprodil (WT+TCN/Ifen, gray square). LTD protocol was delivered at the time indicated by the horizontal bar. **(B)** Averaged LTD magnitude during the last 10 min of recording for WT (patterned bar), WT+Ifen (black bar), WT+TCN (open bar) and WT+TCN/Ifen (gray bar). One-way ANOVA (*F*_(3,25)_ = 1.054, *p* = 0.3863) followed by Dunnett’s *post hoc* test (*p < 0.01) vs. WT. **(C)** NMDAR-LTP obtained in slices from Panx1-KO in the absence (KO, dashed green line) or the presence of Ifenprodil (KO+Ifen, green circle), TCN-201 (KO+TCN, open circle) or TCN-201 plus ifenprodil (KO+TCN/Ifen, light green square). **(D)** Averaged LTD magnitude during the last 10 min of recording for KO (patterned bar), KO+Ifen (green bar), KO+TCN (open bar) and KO+TCN/Ifen (light green bar). One-way ANOVA (*F*_(2,17)_ = 6.368, *p* = 0.0086) followed by Dunnett’s *post hoc* test (*p < 0.01) vs. KO. **(E)** Percentage of LTD blockade for WT and KO slices in the presence of GluN2 antagonists. One-way ANOVA (*F*_(3,14)_ = 41.13, *p* < 0.0001) followed by Tukey’s *post hoc* test (*p < 0.05). The values in parentheses indicate the number of hippocampal slices (left) and the number of animals (right) used. *p < 0.05; ***p < 0.001; ns, non-significant.

**Figure 3:**
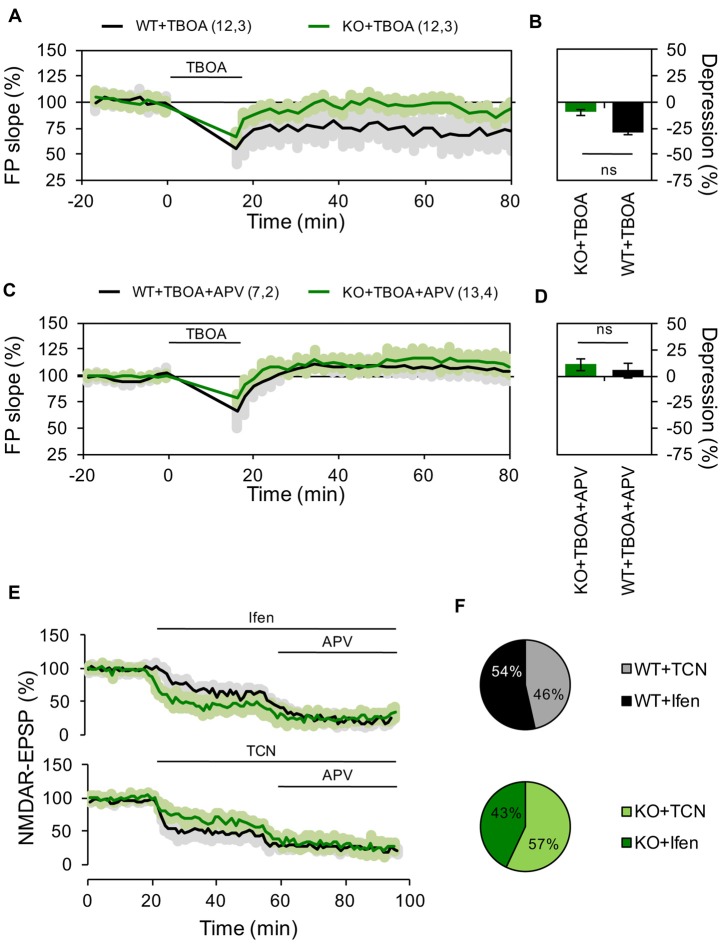
Reduced contribution of extrasynaptic NMDARs to hippocampal LTD in Panx1 deficient mice. **(A)** LTD induced by LFS in the presence of 10 μM DL-TBOA in slices from WT (WT+TBOA, black line) or Panx1-KO (KO+TBOA, green line). LTD protocol and DL-TBOA application were delivered at the time indicated by the horizontal bar. **(B)** Averaged LTD magnitude during the last 10 min of recording for WT (black bar) and KO (green bar). Two-tailed *t*-test revealed non-significant differences. **(C)** LTD induced by LFS in the presence of DL-TBOA and 100 μM APV in slices from WT (WT+TBOA, black line) or Panx1-KO (KO+TBOA, green line). LTD protocol and DL-TBOA application were delivered at the time indicated by the horizontal bar. **(D)** Averaged LTD magnitude during the last 10 min of recording for WT (black bar) and KO (green bar). Two-tailed *t*-test revealed non-significant differences. **(E)** Effects of GluN2 antagonists on NMDAR-EPSPs for WT (black lines) and KO (green lines). Ifen, TCN and DL-APV were applied at the time indicated by the horizontal bar.** (F)** Averaged percentage of GluN2 blockade for WT (top pie chart) and KO (bottom pie chart). Two-tailed *t*-test revealed non-significant differences. The values in parentheses indicate the number of hippocampal slices (left) and the number of animals (right) used. ns, non-significant.

**Figure 4:**
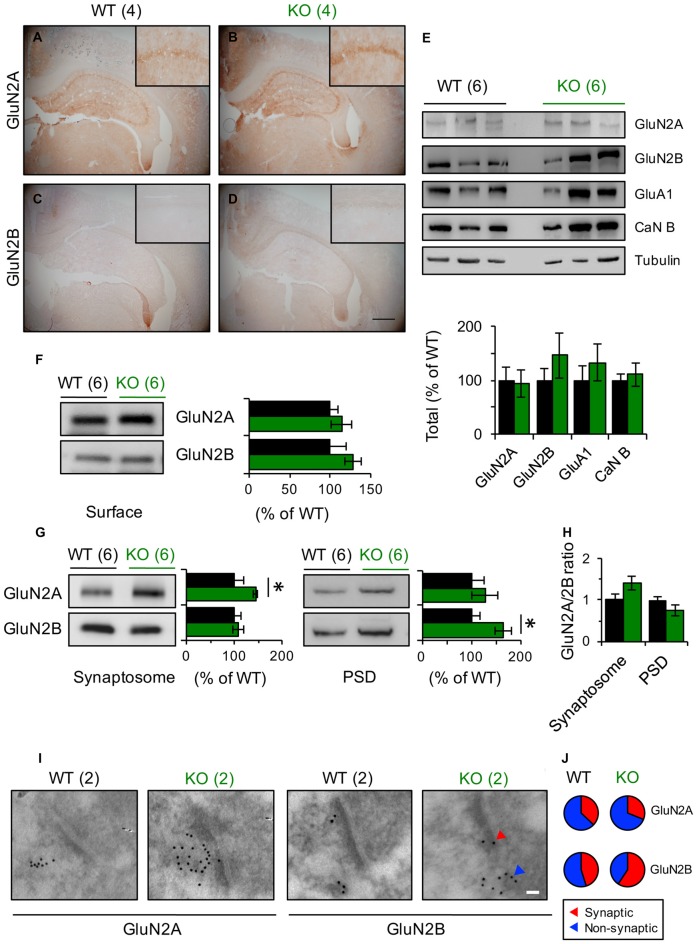
GluN2 subunits expression is modified in Panx1-KO mice.** (A–D)** Representative photomicrographs showing immunohistochemical staining of GluN2A **(A,B)** and GluN2B **(C,D)** in coronal section of WT and Panx1-KO. None differences were observed between WT and Panx1-KO mice. Magnifications of CA1 are superimposed in each photograph. Scale bar represents 500 μm.** (E)** Representative immunoblots (top) and quantification (bottom) for synaptic proteins in hippocampal lysates from WT and Panx1-KO. Two-tailed *t*-test revealed non-significant differences. **(F)** Representative immunoblots (right) and quantification (left) for surface biotinylated GluN2 subunits in hippocampal slices from WT and Panx1-KO. Two-tailed *t*-test revealed non-significant differences. **(G)** Representative immunoblots and quantification for GluN2 subunits in hippocampal synaptosomal (left) and post-synaptic densities (PSD)-enriched fractions (right) from WT and Panx1-KO. Unpaired two-tailed *t*-test (*p = 0.0309 for GluN2A in synaptosomal fraction; *p = 0.0242 for GluN2B in PSD fraction). **(H)** Relative ratio of GluN2A and GluN2B subunits in synaptosomal and PSD fractions. Unpaired two-tailed *t*-test revealed non-significant differences. **(I)** Representative electron micrograph of hippocampal synapses from WT and Panx1-KO mice showing immunogold labeling of GluN2A and GluN2B subunits (Scale bar, 50 nm). **(J)** Percentage of GluN2 particles localized at synaptic (red) and non-synaptic (blue) sites. The values in parentheses indicate the number of animals used.

**Figure 5:**
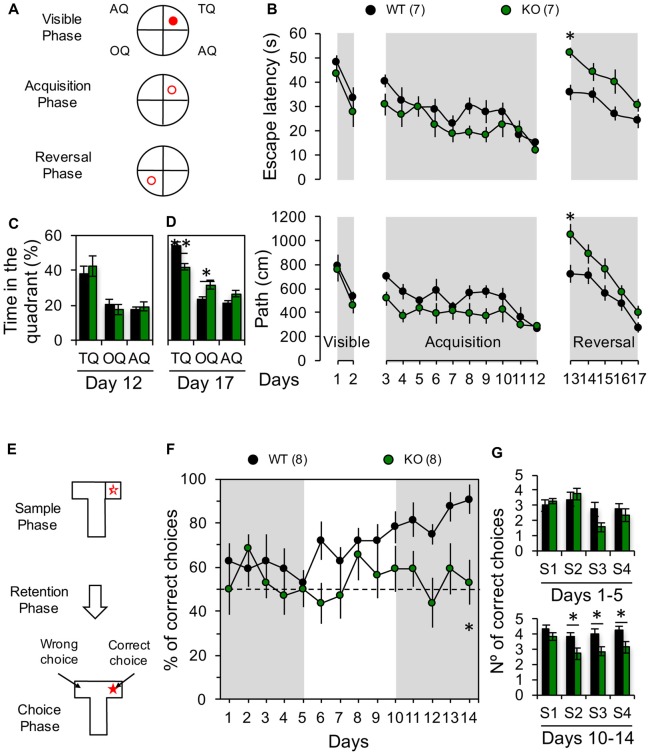
Impaired behavioral flexibility in Panx1-KO mice. **(A)** Diagrams showing the location of the platform (red circle) during visible phase (visible platform in the training quadrant, TQ), acquisition phase (hidden platform in the TQ) and reversal phase (hidden platform in the opposite quadrant, OQ). **(B)** Average latency (top) and distance traveled to the platform (bottom) during all phases of Morris water maze (MWM) test. Two-way ANOVA (*F*_(16,204)_ = 11.60, *p* < 0.0001) followed by Bonferroni *post hoc* test (*p < 0.05). **(C)** Average of time spent in the TQ, OQ, and the averaged adjacent quadrants (AQ) at the end of the acquisition phase (Day 12). Unpaired two-tailed *t*-test (*p* = 0.5562 for TQ; *p* = 0.4299 for OQ; *p* = 0.5353 for AQ). **(D)** Average of time spent in the TQ, OQ, and the averaged AQ at the end of the reversal phase (Day 17). Unpaired two-tailed *t*-test (**p = 0.0024 for TQ; *p = 0.0187 for OQ). **(E)** Schematic of the delayed non-match to place (DNMTP) protocol in a T-maze. **(F)** Percentage of correct choices per day. Two-way ANOVA (*F*_(1,196)_ = 24.63, *p* < 0.0001) followed by Bonferroni *post hoc* test (*p < 0.05). **(G)** Average of correct choices recorded per session during days 1–5 and 10–14. Unpaired two-tailed *t*-test (*p = 0.0256 for S2; *p = 0.0448 for S3; *p = 0.0323 for S4) during days 10–14. The values in parentheses indicate the number of animals used.

### Electrophysiology

Hippocampal slices were prepared as we previously reported (Ardiles et al., 2012). Six to 9-month-old mice were deeply anesthetized with isoflurane, and their brains were quickly removed. Slices (350 μm) were obtained in ice-cold dissection buffer using a vibratome (Leica VT1200S, Leica Microsystems, Nussloch, Germany). Synaptic responses were evoked by stimulating the Schaffer collaterals with 0.2 ms pulses delivered through concentric bipolar stimulating electrodes, and recorded extracellularly in the stratum radiatum of CA1. LTP was induced using four theta burst stimulation (TBS; 10 trains of four pulses at 100 Hz; 5 Hz inter-burst interval) delivered at 0.1 Hz. LTD was induced using low frequency stimulation (LFS; 900 pulses delivered at 1 Hz) in the presence or absence of the glutamate transport inhibitor DL-Threo-β-Benzyloxyaspartic acid (DL-TBOA, 10 μM). LTP and LTD magnitude were calculated as the average (normalized to baseline) of the responses recorded 50–60 min after conditioning stimulation. TCN-201 (TCN, 10 μM) or Ifenprodil (Ifen, 5 μM) were used to block GluN2A- and GluN2B-containing NMDARs respectively (Izumi et al., 2006; Izumi and Zorumski, 2015). Synaptically evoked NMDAR EPSPs were isolated by application of CNQX 10 μM in ACSF containing 2 mM calcium and 0.1 mM magnesium. After 30 min of CNQX pre-incubation, NMDAR-EPSPs were recorded for additional 1 h in the presence of GluN2 subunit antagonist (Ifen or TCN) or the unspecific NMDA receptor antagonist DL-APV 100 μM.

### Histological Studies

For immunohistochemistry analysis, mice were transcardially perfused with 4% paraformaldehyde (PFA) in phosphate buffer pH 7.4 and 20 μm brain sections were obtained using a cryostat (Leica CM1900). Tissue sections were treated with 3% hydrogen peroxide in distilled water for 20 min to block endogenous peroxidase activity. Subsequently, sections were treated with CAS-Block (Invitrogen, 008120) during 30 min followed by a blocking solution to avoid unspecific antibody binding (5% goat serum, Tween 20 and 0.3% PBS 0.1 M) for 30 min. The sections were incubated with mouse primary anti-GluN2A/NR2A clone N32/95 and anti-GluN2B/NR2B clone N59/20 (1:100; NeuroMab), antibodies in blocking solution overnight (ON) at 4°C. The sections were then incubated with HRP-linked secondary goat anti-mouse antibody (1:250; Thermo Fisher Scientific, G21040) for 1 h. Peroxidase reaction was visualized using 3-amino-9-ethylcarbazole (AEC) substrate chromogen system (Vector Laboratories, SK-4800) for 6 min at RT. Finally, tissues were coverslipped with hydrophilic mounting medium (Aquatex, Merck Millipore, 108562). Additionally, to verify the specificity of the secondary antibody, negative controls were performed in parallel omitting the primary antibody. For electron microscopy analysis, mice were transcardially perfused with a mixture of 4% PFA and 0.5% glutaraldehyde, followed by post-fixation in the same mixture ON at 4°C. Brain tissue blocks were trimmed in the CA1 area of the hippocampus and dehydrated in a graded series of ethanol, infiltrated in 1:1 volumes of 100% ethanol and 100% LR White (EMS) during 4 h, immersed in 1.5% OsO_4_ in 0.1 M sodium phosphate buffer (pH 7.4) for 2 h and then embedded in epoxy resin which was polymerized at 50°C ON. Ultrathin sections (90 nm) were made using an ultramicrotome (Leica Ultracut R, Leica Microsystems, Nussloch, Germany) and contrasted with 1% uranyl acetate and lead citrate and located on nickel 300 mesh grids (Ted Pella Inc., Redding, CA, USA). Grids were observed under a transmission electron microscope Philips Tecnai 12 operated at 80 kV (FEI/Philips Electron Optics, Eindhoven) equipped with a digital micrograph camera (Megaview G2, Olympus). For immunogold post-embedding labeling, grids were washed in TBST (Tris buffer solution, 0.1% Triton-X), blocked in a solution of 2% (wt/vol) BSA in TBST then incubated ON in primary antibodies (1:50 mouse GluN2A and GluN2B), washed in TBST, and incubated for 2 h in 12 nm gold bead-conjugated secondary antibody (1:20, Jackson Immunoresearch). Gold particles were counted in serial sections (six sections per series), 15–20 fields per series in two animals. An average 2–3 synapses per field and 4–6 gold particles per synapse were counted.

### Biotinylation

Surface biotinylation was performed in acute hippocampal slices as previously reported (Ardiles et al., 2014). Slices were briefly preincubated in ACSF at 30°C for 1 h, washed twice with ice-cold ACSF and then incubated with sulfo-NHS-SS-Biotin (Thermo Scientific; 1 mg/ml in ACSF) for 45 min on ice with gentle rotation. Excess biotin was removed by means of two brief washes with 10 mM lysine (in ACSF) and two ACSF washes. Slices were then lysed in 500 μl of lysis buffer, centrifuged at 14,000 rpm for 5 min at 4°C and supernatants were discarded. Pellets were resuspended in lysis buffer and biotinylated cell-surface proteins were precipitated with high capacity neutravidin agarose resin (Thermo Scientific, Rockford, IL, USA) and the mixture was rotated ON at 4°C. After several washes with lysis buffer, precipitates were collected by centrifugation (14,000 rpm for 1 min) and detected by immunoblot.

### Synaptosomal Fractionation

Synaptosomes were extracted from hippocampus of adult male mice. Hippocampi were homogenized in ice-cold homogenization buffer (320 mM sucrose, 5 mM Tris, and 0.5 mM EGTA, pH 7.4; protease and phosphatase inhibitor’s cocktails) using a Dounce Tissue Grinder. The homogenate was centrifuged at 1800 rpm for 10 min at 4°C (Beckman F0630 rotor) obtaining a supernatant (S1) which was collected whereas the pellet (P1) was discarded. Then, S1 was centrifuged at 15,000 rpm for 20 min at 4°C (Beckman S4180 rotor). The obtained pellet (P2) containing the membrane proteins was resuspended in homogenization buffer, layered on the top of a discontinuous sucrose density gradient (0.32/1.0/1.2 M) and subjected to ultracentrifugation at 43,000 rpm (Beckman SW-60ti rotor) for 65 min at 4°C. Afterwards, both the sediment and sucrose 0.32/1 M interface were discarded, whereas material accumulated at the interface of 1.0 M and 1.2 M sucrose containing synaptosomal fraction was collected (SP1). SP1 was diluted with lysis buffer to restore the sucrose concentration back to 320 mM and remained on ice with gently agitation for 30 min. Then, SP1 was centrifuged at 15,000 rpm for 30 min. The pellet obtained (PS1) was resuspended in a gradient load buffer, loaded on 0.32/1.0/1.2 M discontinuous gradient and centrifuged at 43,000× rpm for 65 min. The sucrose 1.0/1.2 M interphase, synaptosome fraction 2 (SP2), was recovered and delipidated in delipidating buffer. Next, SP2 was diluted with filling buffer to restore the sucrose concentration and then centrifuged at 15,000 rpm for 1 h. The sediment obtained (PS2) was washed with 50 mM HEPES-Na and centrifuged at 43,000 rpm for 10 min. The final sediment obtained (PS3), containing post-synaptic densities (PSD), was resuspended in 50 mM HEPES-Na and homogenized. PS2 or PSD fractions were quantified for protein concentration as below indicated.

### Immunoblotting

Total proteins and synaptosomal fractions were run on gradient, denaturing gels, blotted, and probed with appropriate antibodies. Samples for total tissue proteins were homogenized in ice-cold lysis buffer (150 mM NaCl, 10 mM Tris-HCl, pH 7.4, EDTA 2 mM, 1% Triton X-100 and 0.1% SDS), supplemented with a protease and phosphatase inhibitor cocktail (Thermo Scientific, Rockford, IL, USA) by using a homogenizator. Protein samples were centrifuged twice for 10 min at 12,000 rpm at 4°C. Protein concentration was determined with the Qubit^®^ Protein Assay Kit (Thermo Scientific, Rockford, IL, USA). For both cases 40 μg of protein per lane were resolved by 10% SDS-PAGE, followed by immunoblotting on PVDF membranes (BioRad, CA, USA) with mouse anti-GluN2A/NR2A clone N32/95, anti-GluN2B/NR2B clone N59/20 (1:500; NeuroMab), anti-calcineurin (1:500; R&D Systems), anti-GluA1 (1:100; Santa Cruz Biotechnology) and anti-Tubulin (1:1000; Santa Cruz Biotechnology). Band intensities were visualized using an enhanced chemiluminescence kit (ECL, BioRad, Hercules, CA, USA) and the intensity of each band was scanned and densitometrically quantified using ImageJ (version 1.46r; NIH, Bethesda, MD, USA). Selected bands were background subtracted using a toolbox provided by ImageJ. Total and biotinylated protein data were normalized to β-tubulin levels and expressed as a % of control group (wild type, WT). For synatosomal proteins, data were normalized to averaged control group (WT) and expressed as % of WT.

### Statistics

All data were presented as mean ± standard error or deviation of the mean (SEM or SD). Data analysis was carried out using the Prism software (GraphPad Software Inc., San Diego, CA, USA). The Kolmogorov–Smirnov test was used to determine whether the data sets were normally distributed. Specific tests used were as follow: the two-tailed *t*-test for two groups comparison and one way-ANOVA test followed by Dunnett’s or Tukey *post hoc* test for multiple comparisons. *p* values < 0.05 were considered to represent significant differences.

## Results

### Absence of Panx1 Modifies the Contribution of GluN2-Containing NMDARs to Synaptic Plasticity

Recently, we have shown that in mice lacking Panx1 or after pharmacological blockade of these channels the threshold for plasticity induction at the Sch-CA1 synapse of adult animals is dynamically modified (Ardiles et al., 2014), but the molecular mechanisms involved remain unclear. Previous studies have suggested that the subunit composition of NMDARs can control the polarity of synaptic plasticity (Shipton and Paulsen, 2014). Therefore, we analyzed if an altered NMDARs composition could cause the differences in the expression of plasticity observed in adult Panx1-KO mice. For testing this we analyzed the contribution of subunit-specific NMDARs on the induction of either LTP or LTD, using a TBS protocol or a low frequency stimulation protocol respectively (see “Materials and Methods” section), at the Sch-CA1 pathway of age-matched adult KO and WT animals (Figures 1, 2). Confirming previous results (Ardiles et al., 2014), a TBS protocol induced a stronger potentiation in Panx1-KO animals compared to WT animals (51 ± 6.1% and 105.4 ± 9.76% for WT and KO animals, *n* = 14–18 slices, Figure 1). Inhibition of GluN2A- and GluN2B-containing NMDARs, by preincubation of slices with either 10 μM TCN-201 (TCN) or 5 μM Ifenprodil (Ifen) respectively, generated a reduction in the magnitude of LTP both in WT and KO animals confirming that LTP under these conditions depends on NMDARs (33.4 ± 6.75% and 3.5 ± 0.48% potentiation with Ifen incubation for WT an KO animals respectively; 15.1 ± 3.26 and 31.8 ± 4.81% potentiation with TCN incubation). As expected, Ifen together with TCN almost completely reduced LTP in both WT and KO (7.3 ± 7.34% and 10.8 ± 8.6% potentiation for WT and KO animals respectively). Remarkably, we observed differences in the relative effects of the specific NMDARs antagonists on the LTP magnitude in WT and KO mice (Figure 1E). While the GluN2A-specific antagonist produced a similar reduction in LTP in both groups (70.27 ± 4% and 69.82 ± 5% reduction of control LTP with TCN preincubation for WT and KO mice, *n* = 4 slices), the GluN2B antagonist was significantly more efficient in reducing potentiation in KO animals (34.25 ± 7% and 96.68 ± 2% reduction of control LTP with Ifen preincubation for WT and KO mice, *n* = 5 slices). These results suggest that the lack of Panx1 changes the contribution of GluN2 subunits to hippocampal LTP, enhancing the participation of GluN2B.

Next, we examined if similar modifications occur during LTD. As we previously reported (Ardiles et al., 2014), a LFS protocol effectively evoked enduring LTD in hippocampal slices of WT mice, but induced a robust LTP in hippocampal slices from KO mice (22.5 ± 2.84% depression and 17.1 ± 7.25% potentiation for WT and KO animals, *n* = 9–12 slices, Figure 2). In control mice, blocking either GluN2A or GluN2B containing receptors produced a mild decrease in the depression of synaptic transmission induced by LFS (14.36 ± 2.84% and 15.85 ± 2.95% depression for TCN and Ifen treatment respectively, *n* = 5–7 slices; Figures 2A,B). Surprisingly, in mice lacking the Panx1 channel, blocking GluN2B-containing NMDARs reversed the KO phenotype and a robust LTD was observed after LFS (16.01 ± 5.16% depression with Ifen treatment, Figures 2A,B), while blocking GluN2A-containing NMDARs indicated a trend toward an increase in the LTP observed in the absence of antagonists (37 ± 8.16% potentiation with TCN treatment, Figures 2A,B). The combination of GluN2 subunit antagonists precluded LFS-induced synaptic changes in both WT and KO (5.8 ± 5.1% and 3.7 ± 6.6% potentiation respectively). Therefore, while both subunits contribute equally to LTD in WT animals (36.03 ± 3% or 29.4 ± 7.25% reduction of control LTD with TCN or Ifen incubation respectively, *n* = 5–7 slices; Figure 2E), GluN2A NMDAR subunits seem to play a major role in LTD of KO animals compared to WT, since it can induce LTD in the absence of GluN2B-containing NMDARs, which in contrast favor potentiation (Figures 2C,D).

It has been reported that LFS-induced LTD is more difficult to evoke in older than younger animals (Errington et al., 1995), but that manipulations increasing glutamate spillover facilitate LTD induction in adult animals through the activation of extrasynaptic GluN2B-containing NMDARs (Massey et al., 2004; Wong et al., 2007; Duffy et al., 2008). To investigate how extrasynaptic NMDARs might affect the differential effect of LFS in WT and KO mice, we induced LTD in the presence of the glutamate transport inhibitor DL-TBOA (10 μM). As expected, TBOA application facilitated the induction of LTD in WT animals (28.5 ± 2.84% depression, *n* = 12 slices, Figures 3A,B). In contrast, in KO animals TBOA application during LFS precluded the expression of LTP and even a slight depression was observed (10 ± 5.1% depression, *n* = 12 slices, Figures 3A,B). Preincubating hippocampal slices with the NMDAR antagonist APV (100 μM; Figures 3C,D) blocked LTD in both WT and KO groups, confirming that the observed plastic processes depended on the participation of NMDARs.

To further evaluate if Panx1 ablation modifies the expression of different NMDARs subtypes, we examined the effect of selective GluN2 subunit antagonists on NMDAR-mediated EPSPs evoked by stimulation of the Schaffer collateral. Notably, application of Ifen had a greater effect on NMDAR-EPSP in the KO group (46% and 57% of reduction in WT and KO respectively), while TCN produce a bigger reduction of NMDAR-EPSP in WT animals (54% and 43% of reduction in WT and KO respectively; Figures 3E,F). Together, these results suggest that Panx1-deficiency affects the contribution of specific GluN2 subunits to hippocampal excitatory LTP/D.

### Synaptic GluN2 Subunit Composition Is Altered in the Absence of Panx1

How can the differential contribution of subunit-specific NMDARs to plasticity in Panx1-KO and WT mice be explained? Modulation of NMDARs expression or trafficking is known to influence the manifestation of plastic phenomena. Immunohistochemical labeling of hippocampal slices showed no difference in the expression of GluN2A or GluN2B subunits between KO and WT animals (Figures 4A–D). Similarly, western blot analysis of hippocampal homogenates showed comparable expression of the studied NMDAR subunits between these two animal cohorts (Figure 4E). Moreover, biotinylation assay in hippocampal slices demonstrated that the total content of GluN2 subunits at the plasma membrane was also unaltered (Figure 4F). Nevertheless, when we measured the amount of GluN2A and GluN2B subunits at the subcellular level by using specific membrane fractions, we observed that they tended to be more concentrated in synaptosomal- and PSD-enriched fractions obtained from KO mice. Indeed, GluN2B protein was significantly enriched in PSD fractions whereas GluN2A was significantly enriched in synaptosomes of KO compared to WT (Figure 4G). Interestingly, estimating the GluN2 subunit ratio, we observed that GluN2A/GluN2B tended to be higher in synaptosomes and lower in PSD fractions of the KO group compared to WT animals, although these tendencies were not statically significant (Figure 4H). Despite that, these observations suggest that the synaptic (PSD fraction) vs. non-synaptic (synaptosomal fraction without PSD) GluN2 subunit content is differentially modulated by the Panx1 channel.

To further examine the synaptic distribution of GluN2 subunits, we performed electron-microscopy immunogold detection of GluN2A and GluN2B proteins in hippocampal slices (Figures 4I,J). In general, we found that the total number of GluN2 gold particles per synapse did not differ across genotype (6.5 ± 1.56 and 4.4 ± 0.75 gold particles for WT and KO synapses, *n* = 6–12 sections Figure 4I). Although in both, WT and KO samples, GluN2A particles was mainly extrasynaptically located, in KO animals we observed that GluN2B particles tended to accumulate in synaptic membranes while in WT animals they were homogeneously distributed (Figure 4I).

Collectively, these results suggest that the absence of Panx1 modifies the content of GluN2A- and GluN2B-containing NMDARs in the synaptic membrane and hence could explain the different contribution of GluN2 subunits to LTP/LTD and the shift in the induction of NMDAR-dependent synaptic plasticity (Ardiles et al., 2014).

### Panx1-KO Mice Exhibits Deficits in Flexibility of Hippocampal-Dependent Spatial Memory

LTD play important roles in several forms of learning and memory, particularly those involving the modification of previously acquired information and the processing of newly learned memories (Collingridge et al., 2010). For instance, hippocampal LTD has been related with the perception of novelty during object recognition (Manahan-Vaughan and Braunewell, 1999; Kemp and Manahan-Vaughan, 2004) or during spatial object recognition memory tasks (Goh and Manahan-Vaughan, 2013). Moreover, LTD has been demonstrated to be necessary for the consolidation of fear memory (Liu et al., 2014) and spatial memory (Ge et al., 2010), and the behavioral flexibility of spatial learning (Nicholls et al., 2008; Dong et al., 2013; Mills et al., 2014). As we have previously shown that removing the Panx1 gene or blocking Panx1 channels prevents the induction of hippocampal NMDAR-LTD (Ardiles et al., 2014), we wanted to evaluate if this also results in a deficit in spatial memory flexibility. To this end we first used MWM to evaluate the acquisition and the reversion of spatial learning (Figures 5A–D). First, we trained the animals to find a visible platform, and found that both groups learned to find the position of the platform at comparable levels (days 1–2; Figure 5B), showing similar escape latencies and traveled distance at the end of this phase (day 2; latency: WT: 531.2 ± 50.2 s, *n* = 7 and KO: 461.9 ± 63.08 s, *n* = 7; path: WT: 33.8 ± 4.1 s, *n* = 7 and KO: 27.9 ± 6.2 s, *n* = 7; Figure 5B). Next, we trained animals to find a hidden platform placed at a fixed quadrant of the pool assisted by visual clues (days 3–12; Figure 5B). Both animal groups learned to find the hidden platform at similar rates during the acquisition phase (Figure 5B), although KO mice showed a trend for a better performance, during the first days of this acquisition phase, although not statistically significant, suggesting that KO mice can learn quicker than WT. Nevertheless, both groups showed similar path length and escape latencies at the end of the acquisition phase (day 12; latency: WT: 271.8 ± 41.1 s, *n* = 7 and KO: 284.3 ± 44.01 s, *n* = 7; path: WT: 15.5 ± 1.4 s, *n* = 7 and KO: 12.3 ± 1.7 s, *n* = 7; Figure 5B) pointing towards a similar level of task-learning for both groups. We then trained the animals to find a hidden platform in the OQ to the previously learned location (reversal phase; Figure 5B), and evaluated the acquisition of this new spatial memory (Figure 5B). Although both groups exhibited increased path length and escape latencies during the first days, ultimately all animals learned to find the new location of the platform (Figure 5B). Remarkably, on the first day of the reversal phase (day 13), the KO group showed a significantly greater path length compared to the WT group indicating that reverse learning took a longer time in KO animals (day 13; latency: WT: 732.9 ± 74.1 s, *n* = 7 and KO: 1060.4 ± 81.6 s, *n* = 7; path: WT: 35.7 ± 3.2 s, *n* = 7 and KO: 51.9 ± 2.0 s, *n* = 7; Figure 5B). Moreover, a trend to perform worse was maintained during all sessions of the reversal phase for KO animals (days 13–17; Figure 5B). Accordingly, at the end of the reversal phase, these mice spent significantly more time in the OQ whereas WT displayed a longer preference for the training quadrant (TQ) (Figure 5C). These results indicate that ablation of the Panx1 gene decrease the capability of rapidly adjusting old and creating new spatial associations. To further verify the effects of Panx1 KO on learning flexibility, we used another hippocampus-dependent memory test, a T-maze DNMTP test, in which animals need to acquire and retain new spatial information each successive trial in which the reward location is changed (McHugh et al., 2008). WT animals progressively improved their performance with each trial starting from the 6th day, reaching almost a 90% of correct choices at the end of the task (day 14, Figure 5F). In stark contrast, KO mice continuously performed only at chance level during the entire experiment (Figure 5F). Interestingly, when we assessed the animal performances by trial we found that KO mice showed a significantly decreased number of correct choices in trials 2, 3 and 4 during the last 5 days of the task, when the inter-trial delay was only 15 min, but in the first trial when delay was 24 h, no differences were observed (Figure 5G). Therefore, these data show that, as with the reversal phase of the MWM, the absence of Panx1 affects the reverse learning of previously acquired spatial information in DNMTP, supporting that behavioral flexibility for hippocampal-dependent spatial memory tests is reduced in Panx1 KO mice.

## Discussion

Recent studies have provided evidences for a novel physiological role of Panx1 channels in the modulation of activity-dependent phenomena such as synaptic plasticity and behavior (Prochnow et al., 2012; Ardiles et al., 2014). In the present study, we have provided evidences that Panx1 ablation modifies the content of synaptic vs. non-synaptic GluN2 subunits of NMDARs and changes the relative contribution of specific GluN2 subunits to LTP and LTD at the Schaffer collateral–CA1 synapses in the adult hippocampus. These modifications lead subsequently to impairments in the balance of synaptic plasticity and cognitive flexibility of spatial learning.

### Behavioral Phenotype of Pannexin 1 Deficient Mice

Since the ablation of Panx1 channels affects the induction of NMDAR-LTD (Ardiles et al., 2014), we predicted that Panx1 absence might disrupt LTD-dependent components of learning and memory such as the acquisition of a new spatial memory. Using MWM and DNMTP tests we examined learning and relearning of a new spatial location (Figure 5). We found that mice lacking Panx1 take more time to learn a new location in the MWM showing longer path and latency to find the platform, spending more time in the previous quadrant than in the new location compared to WT animals. Accordingly, KO mice showed impaired learning on the T maze, performing at chance level over the entire test, whereas WT group gradually improved their performance in this task. These findings show that Panx1-deficient mice exhibit impairment in memory tasks where behavioral flexibility is necessary to find a specific location. Previous studies using a different Panx1-deficient mouse (Panx1^−/−^) revealed that loss of Panx1 leads to enhanced anxiety and impaired object recognition and spatial memory (Prochnow et al., 2012). Consistent with our findings, Prochnow et al. (2012) reported that Panx1^−/−^ mice display an altered ability to discriminate between a known and a new object using the object recognition memory test, indicating that Panx1 loss also affects another behavioral paradigm based in LTD plastic mechanisms (Griffiths et al., 2008). In this regard, considerable evidence indicates that LTD is important to learning and memory behaviors particularly involving the processing and acquisition of new information (Collingridge et al., 2010). For instance, LTD has been shown to regulate novelty acquisition in an object recognition memory (Manahan-Vaughan and Braunewell, 1999; Kemp and Manahan-Vaughan, 2004), spatial object recognition (Goh and Manahan-Vaughan, 2013), working/episodic like memory (Etkin et al., 2006), the consolidation of fear memory (Liu et al., 2014) and spatial memory (Ge et al., 2010). Notably, LTD has been reported to regulate behavioral flexibility (Morice et al., 2007; Nicholls et al., 2008; Kim et al., 2011; Dong et al., 2013; Mills et al., 2014). Moreover, manipulation of LTD signaling, such as genetic deletion or pharmacological inhibition of calcineurin (CaN) and PI3Kγ have been showed to produce deficits in NMDAR-dependent LTD and behavioral flexibility (Kim et al., 2011), further supporting a role of LTD in memory processes.

Together, our findings suggest that Panx1 ablation promotes changes in LTD mechanisms that impact on related behavior.

### Mechanisms of Synaptic Plasticity Modulation by Panx1 Channels

It is still unclear how Panx1 ablation can modulate synaptic and cognitive functions. For instance, it is unknown whether Panx1 exerts this function directly or through the interaction with other synaptic proteins. Panx1 might closely interacts with NMDAR GluN2A and GluN2B subunits, since it has been suggested that these channels can be activated by NMDAR stimulation (Thompson et al., 2008). Therefore, it is possible that changes in the expression or localization of specific GluN2 subunits of NMDARs could explain the modification in the induction of synaptic plasticity observed in KO mice (Ardiles et al., 2014). In fact, GluN2B subunits are more important for LTP induction than GluN2A in KO animals, while the opposite is observed in the WT group. At the same time, LFS-induced LTD was absent in KO animals under GluN2A subunit blockade (i.e., GluN2B activation), instead a potentiation was induced (Figure 2), indicating that GluN2B-containing NMDARs rather promote LTP over LTD. Interestingly, when GluN2B was blocked (i.e., GluN2A activation) a similar LTD was obtained in WT and KO mice indicating that GluN2A containing NMDARs support LTD in both groups (Figure 2). Moreover, we also found that LFS applied in the presence of TBOA had a lower effect in slices from KO mice, suggesting that the contribution of extra-synaptic GluN2B-containing NMDARs to LTD is lower in KO compared to WT (Figure 3A).

GluN2A and GluN2B are the main GluN2 subunits expressed in the adult rodent brain, particularly in cerebral cortex and hippocampus, and they have key roles in synaptic function and plasticity (Yashiro and Philpot, 2008). The identity of GluN2 subunits determines some of the biophysical properties of NMDARs important for synaptic plasticity. For instance, GluN2A-containing receptors show lower affinity for glutamate, higher sensitivity to Mg^2+^ blockade, greater channel open probability and Ca^2+^ desensitization than GluN2B (Yashiro and Philpot, 2008). This has important implications because these properties can determine the responses to stimulation at different frequencies and hence the directionality of the synaptic modifications (Erreger et al., 2005). In other words, at low frequencies typically used to induce LTD (1 Hz), GluN2B makes a larger contribution to the total charge transfer and calcium influx than GluN2A (Erreger et al., 2005). However, under high-frequency stimulation, typically used to induce LTP (100 Hz), the current mediated by GluN2A considerably exceeds that of GluN2B (Erreger et al., 2005). Currently, there is contrasting evidence regarding the differential roles of GluN2 subunits to LTP and LTD induction. For instance, it has been speculated that GluN2B favors more LTP induction than GluN2A (Tang et al., 1999; Hendricson et al., 2002; Wong et al., 2002; Philpot et al., 2003; Barria and Malinow, 2005; Bartlett et al., 2007; Morishita et al., 2007; Philpot et al., 2007; Wang et al., 2009). However, it has also been reported that GluN2A-, but not GluN2B-containing receptors, mediate LTP (Liu et al., 2004; Massey et al., 2004). On the other hand, some reports indicate that induction of LTD does not require GluN2B-containing NMDARs activation (Hendricson et al., 2002; Bartlett et al., 2007; Morishita et al., 2007). Indeed, a LFS protocol (900 pulses, 1 Hz) induces LTP instead LTD in a GluN2A KO mice (Bartlett et al., 2007). In contrast, other evidences suggest that GluN2B but not GluN2A subunits are important for LTD (Liu et al., 2004; Kollen et al., 2008). It should be noted that the contribution of these GluN2 subunits also depends on the developmental, regional and behavioral experience context in which they are studied. Notwithstanding, these evidences suggest that the threshold for the induction of LTD and LTP is governed by the ratio of GluN2A/GluN2B (Yashiro and Philpot, 2008) and alterations in this ratio might generate, in turn, impairments in synaptic plasticity and brain mechanisms underlying learning and memory. Thus, it has been proposed that synapses exhibiting a high GluN2A/GluN2B ratio, would favor the induction of LTD, while synapses with a low GluN2A/GluN2B ratio would be more prone to express LTP (Yashiro and Philpot, 2008). Our results suggest that Panx1 loss might modify GluN2A/GluN2B ratio which generates the observed changes in LTP and LTD expression. Indeed, we found that the synaptic content of GluN2B-subunit of NMDAR was significantly increased in hippocampal PSD-enriched fractions, whereas GluN2A subunit was significantly increased in synaptosomes obtained from KO mice compared to WT animals. Although comparison between groups revealed no significant differences in the GluN2A/GluN2B ratio in PSD and synaptosomal fractions, we observed a tendency to a reduction in the ratio in KO PSDs compared to WT. The latter suggests that GluN2B levels are higher than GluN2A in KO PSDs. On the contrary, in KO synaptosomes the ratio tended to be higher than WT, suggesting in this case that GluN2B levels are lower than GluN2A in KO synaptosomes. These assumptions were supported by visualization of hippocampal Sch-CA1 synapses by electron microscopy, where we observed a redistribution of gold particles positive for GluN2A- and GluN2B-subunits (Figures 4I,J). It is noteworthy that in KO synapses, most of gold particles positive to GluN2A subunits accumulate in non-synaptic sites, whereas GluN2B particles localize overlying spine membrane.

According with these results, we observed a lower contribution of extra-synaptic GluN2B-containing NMDARs to LTD, revealed by TBOA incubation, in KO slices compared to WT animals (Figures 3E,F), suggesting a modification either in traffic or expression of GluN2 subunits from/toward non-synaptic sites. Indeed, we found a tendency to greater surface level of both GluN2 subunits in KO mice compared to WT animals (Figure 4F). Nevertheless, we did not found changes in total levels of GluN2 subunits evaluated in hippocampal homogenates (Figure 4E). In agreement with that, an earlier report by Prochnow et al. (2012), using Panx1^−/−^ mice, showed no changes in synaptic plasticity related-genes including GluN2 subunits of NMDARs indicating that expression of GluN2 is not affected.

In summary, our data revealed novel functions of Panx1 channels in synaptic plasticity and memory flexibility. Furthermore, these results are in line with a modification in the LTD mechanism thought to be implied in these cognitive processes. For instance, we found that KO mice display a modification in synaptic proteins, some of which are critical for the induction or expression of LTD. Based on the current findings we propose that Panx1 channels modulate the induction of synaptic plasticity by changing the distribution of subunit-specific NMDARs, ultimately leading to deficiencies in learning and memory processes.

## Author Contributions

IG, CSS, DL-E and ÁOA designed the research. IG, CSS, DL-E, CEstay, CF-M and ÁOA performed experiments, analyzed data and contributed to figures. CElgueta, PM, AMG-J and ADM contributed analytic tools. ÁOA wrote the article.

## Conflict of Interest Statement

The authors declare that the research was conducted in the absence of any commercial or financial relationships that could be construed as a potential conflict of interest.
